# Mitophagy alleviates cisplatin-induced renal tubular epithelial cell ferroptosis through ROS/HO-1/GPX4 axis

**DOI:** 10.7150/ijbs.80775

**Published:** 2023-02-13

**Authors:** Qisheng Lin, Shu Li, Haijiao Jin, Hong Cai, Xuying Zhu, Yuanting Yang, Jingkui Wu, Chaojun Qi, Xinghua Shao, Jialin Li, Kaiqi Zhang, Wenyan Zhou, Minfang Zhang, Jiayi Cheng, Leyi Gu, Shan Mou, Zhaohui Ni

**Affiliations:** 1 Department of Nephrology, Molecular Cell Lab for Kidney Disease, Shanghai Peritoneal Dialysis Research Center, Ren Ji Hospital, Uremia Diagnosis and Treatment Center, Shanghai Jiao Tong University School of Medicine, Shanghai, 200127, China; 2 Department of Nephrology, Shuguang Hospital Affiliated to Shanghai University of Traditional Chinese Medicine, Shanghai 201200, China; 3 Tianping Community Health Service Center, Shanghai, 200031, China

**Keywords:** Acute kidney injury, cisplatin nephrotoxicity, ferroptosis, mitophagy

## Abstract

Cisplatin is widely recommended in combination for the treatment of tumors, thus inevitably increasing the incidence of cisplatin-induced acute kidney injury. Mitophagy is a type of mitochondrial quality control mechanism that degrades damaged mitochondria and maintains cellular homeostasis. Ferroptosis, a new modality of programmed cell death, is characterized by iron-dependent phospholipid peroxidation and oxidative membrane damage. However, the role of mitophagy in ferroptosis in kidney disease is unclear. Here, we investigated the mechanism underlying both BNIP3-mediated and PINK1-PARK2-mediated mitophagy-induced attenuation of ferroptosis in cisplatin-induced acute kidney injury. The results showed that cisplatin induced mitochondrial injury, ROS release, intracellular iron accumulation, lipid peroxidation and ferroptosis in the kidney, which were aggravated in *Bnip3 knockout*, Pink1 *knockout* or *Park2* knockout cisplatin-treated mice. Ferrstatin-1, a synthetic antioxidative ferroptosis inhibitor, rescued iron accumulation, lipid peroxidation and ferroptosis caused by inhibition of mitophagy. Thus, the present study elucidated a novel mechanism by which both BNIP3-mediated and PINK1-PARK2-mediated mitophagy protects against cisplatin-induced renal tubular epithelial cell ferroptosis through the ROS/HO1/GPX4 axis.

## Introduction

Drug-induced acute kidney injury is the second most common cause of acute kidney injury in hospitalized patients, especially in the intensive care unit^1^, ^2^. Among all types of acute kidney injury, drug-induced acute kidney injury accounts for 14-37.5% in several studies 3, 2. Nephrotoxic drugs, such as antibiotics (gentamicin), diuretics (furosemide), chemotherapeutic drugs (cisplatin) and calcineurin inhibitors (tacrolimus), induce acute kidney injury 1. Cisplatin is widely recommended in combination for the treatment of lung cancer 4, bladder cancer 5 and gastric cancer 6. During cisplatin treatment, approximately 20%-30% of patients develop acute kidney injury 7. The mechanism of cisplatin-induced acute kidney injury includes oxidative stress, mitochondrial dysfunction, and endoplasmic reticulum stress, which result in apoptosis, necrosis and ferroptosis of renal tubular epithelial cells, causing rapid loss of kidney function 8-11. Elucidating the precise molecular mechanisms underlying cisplatin-induced acute kidney injury will provide evidence for future treatment.

Mitophagy, a type of specific autophagy that eliminates and degrades damaged mitochondria to ensure the quality control of mitochondria, is essential for cellular homeostasis and mammalian survival 12, 13. There are two major mitophagy pathways, namely, PARK2-dependent and PARK2-independent signaling 14. In the PARK2-dependent pathway, PINK1 activates PARK2 to target many mitochondrial proteins, including NDP52, OPTN and p62, and it combines with LC3 to deliver damaged mitochondria to autophagosomes 15. BNIP3 mediates the PARK2-independent pathway of mitophagy by directly binding with LC3 to initiate mitophagy 15. Mitophagy prevents excessive reactive oxygen species (ROS) accumulation, activates the mitochondrial apoptotic cascade and inhibits mtDNA and damage-associated molecular pattern release, which reduces renal tubular epithelial cell injury and acute kidney injury 16. Our recent studies have confirmed that mitophagy, both PINK1-PARK2-mediated and BNIP3-mediated, protect against contrast-induced acute kidney injury by reducing oxidative stress 17, 18. In cisplatin-induced acute kidney injury, Wang *et al.* showed that the PINK1-PARK2 pathway decreases apoptosis 19. However, BNIP3-mediated mitophagy and the downstream pathway and mechanism are poorly understood in cisplatin-induced acute kidney injury.

Ferroptosis is a new modality of programmed cell death different from apoptosis, necrosis and pyroptosis, and it is characterized by iron-dependent phospholipid peroxidation and oxidative membrane damage 20. Multiple pathways regulate ferroptosis through redox homeostasis, mitochondrial dysfunction and various signaling pathways, such as Nrf2/Keap1 signaling and p53 signaling 21. GPX4 is a key suppressing factor of ferroptosis via the phospholipid hydroperoxide (PLOOH)-neutralizing enzyme 22. Upregulation of HO1 and loss of GPX4 directly inhibit cystine import, block GSH and promote accumulation of PLOOHs, causing rapid and unrepairable damage to membranes and ferroptosis 23, 24. Moreover, the iron-dependent Fenton reaction, which is induced by excessive Fe^2+^ and hydrogen peroxide, increases ROS and PLOOH, further promoting ferroptosis 25. In kidney studies, ferroptosis has been reported to exacerbate ischemia reperfusion or folic acid-induced acute kidney injury 26-29. In addition, several researchers have shown that cisplatin activates ferroptosis, thereby aggravating acute kidney injury 11, 30, 31. Unfortunately, the upstream pathway is not completely known in cisplatin-induced acute kidney injury, and it remains unclear whether autophagy, oxidative stress and mitochondrial dysfunction regulate cisplatin-induced ferroptosis.

Here, we hypothesized that mitophagy protects against cisplatin-induced acute kidney injury by reducing renal tubular epithelial cell ferroptosis. The present study was designed to investigate BNIP3-mediated and PINK1-PARK2-mediated mitophagy and its regulation of ferroptosis in a cisplatin-induced acute kidney injury model using *Pink1*, *Park2* and *Bnip3* knockout mice. The aim of the present study was to provide a new viewpoint for the treatment of chemotherapeutic drug-induced acute kidney injury.

## Results

### 1. Cisplatin induces renal tubular epithelial cell ferroptosis *in vivo* and *in vitro*

**Fig 1:**
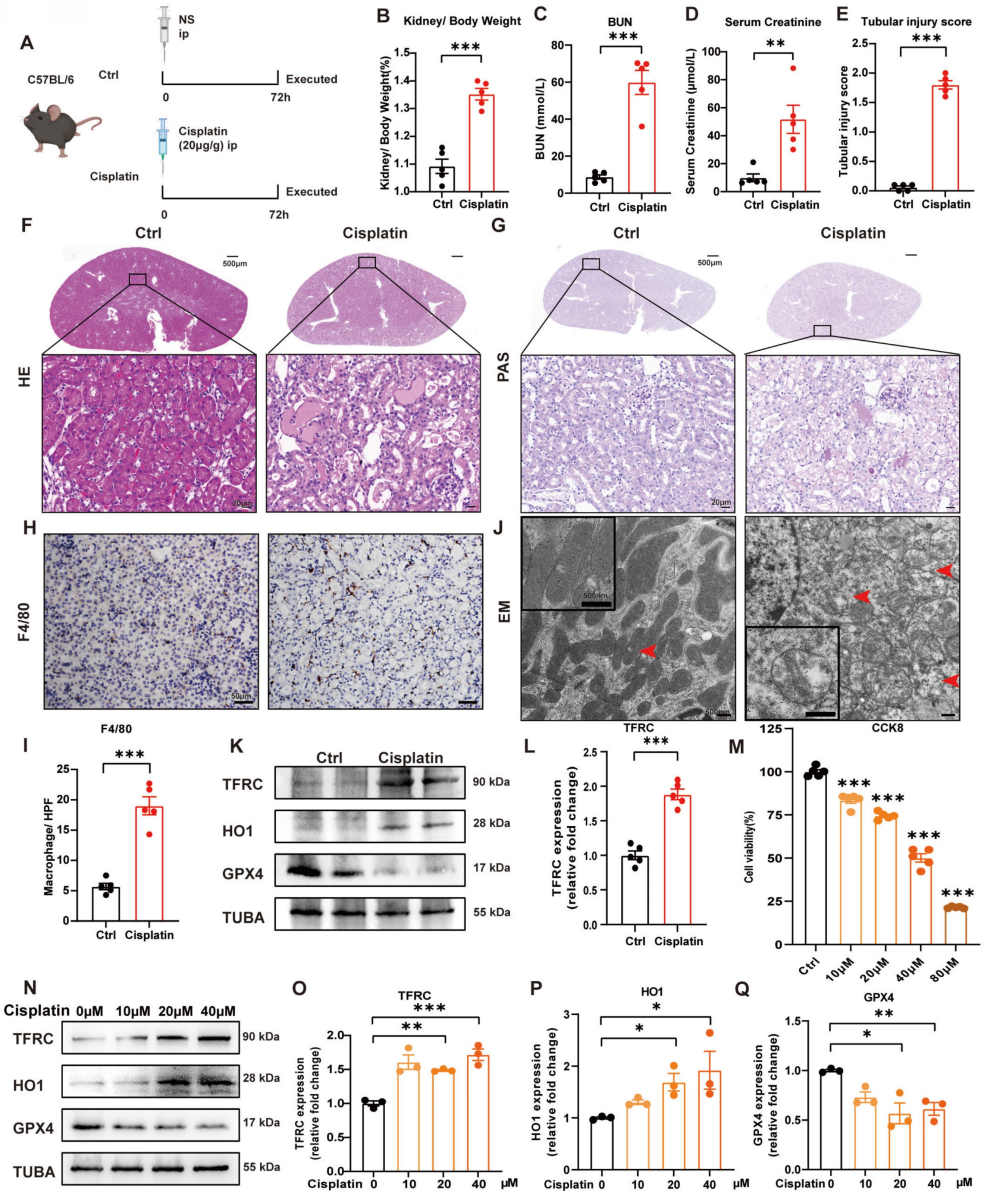
** Cisplatin induces renal tubular epithelial cell ferroptosis *in vivo* and *in vitro*.** (A) Schematic representation of cisplatin-induced acute kidney injury. Briefly, mice were intraperitoneally injected with cisplatin (20 mg/kg) and sacrificed after 72 hours. (B) Kidney/body weight, (C) serum creatinine levels and (D) BUN levels after cisplatin administration. (E-G) Representative HE staining, PAS staining and pathological tubular injury score in the renal cortex. (H, I) Representative IHC images and quantification of F4/80 in the kidney cortex. (J). Representative TEM images of mitochondrial morphology in renal tubular epithelial cells of Ctrl and cisplatin-treated mice. (K, L) Immunoblot analysis of TFRC, HO1, and GPX4 in kidney lysates. (M) CCK-8 assay of HK-2 cells treated with different concentrations of cisplatin for 24 hours. * indicates compared to the Ctrl group. (N-Q) Immunoblot analysis and quantification of TFRC, HO1 and GPX4 in HK-2 cell lysates. Data are presented as the mean ± SEM (n=5 *in vivo*; n=3 *in vitro*). *p<0.05, **p<0.01 and ***p<0.001.

We generated a cisplatin-induced acute kidney injury mouse model as previously described 30, 32. Briefly, male mice (6-8 weeks old and 20-25 g) were intraperitoneally injected with cisplatin (20 mg/kg body weight) and sacrificed after 72 hours (Figure 1A). At the perfusion time, we recorded the body and kidney weights of the mice to calculate the kidney/body weight ratio. After cisplatin treatment, the body weight was significantly decreased, but there was no significant difference in kidney weight (Supplemental Figure 1A-1C), which resulted in an increase in kidney/body weight in the cisplatin group (Figure 1B). The blood urea nitrogen (BUN) and serum creatinine levels were significantly in the cisplatin group, which confirmed that cisplatin induced acute kidney injury* in vivo* (Figure 1C, 1D). Hematoxylin and eosin (HE) and periodic acid-Schiff (PAS) staining showed more cast formation and intraepithelial vacuolar degeneration as well as a higher tubular injury score in cisplatin-treated kidneys compared to Ctrl kidneys (Figure 1E-1G). F4/80, a marker of macrophages, suggested severe inflammatory infiltration after cisplatin injection (Figure 1H, 1I). Transmission electron microscopy (TEM) analysis indicated that cisplatin induced rupture of the outer mitochondrial membrane, disappearance of mitochondrial cristae and vacuolization in mitochondria (Figure IJ, red arrow), which are mitochondrial characteristics of ferroptosis 33, 34. In addition, cisplatin upregulated malondialdehyde (MDA) release and iron levels but downregulated superoxide dismutase (SOD) and glutathione (GSH) levels (Supplemental Figure 1D-1G). Immunoblot analysis showed an increase in transferrin receptor 1 (TFRC) and heme oxygenase 1 (HO1) but a decrease in glutathione peroxidase 4 (GPX4), a specific ferroptosis inhibition protein (Figure 1K, 1L; Supplemental Figure 1H, 1I). These data indicated the presence of cisplatin-induced acute kidney injury, resulting in an increase in ROS, uptake of cellular iron and cell ferroptosis.

In the* in vitro* study, we first used different concentrations of cisplatin in the cell culture medium from 10 to 80 μM and evaluated cell viability by a CCK8 assay (Figure 1M). To further evaluate the function of cisplatin on the transferrin receptor and ferroptosis, we quantified TFRC, HO1 and GPX4 protein expression by immunoblot analysis, which demonstrated that cisplatin induced ferroptosis in the HK-2 cell line (Figure 1N-1Q).

### 2. Mitophagy is increased in cisplatin-induced acute kidney injury

**Fig 2:**
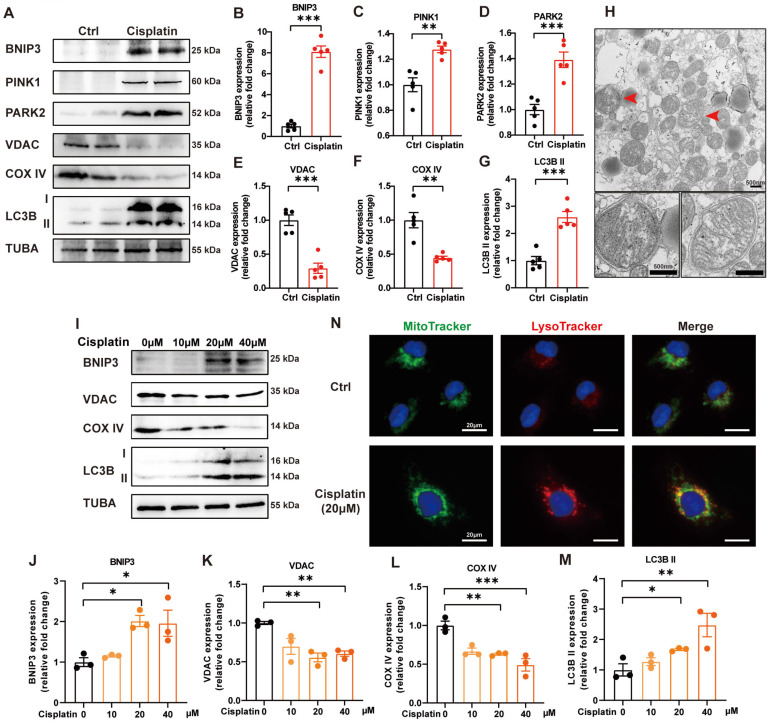
** Mitophagy is increased in cisplatin-induced acute kidney injury.** (A-G) Immunoblot analysis and quantification of BNIP3, PINK1, PARK2, VDAC, COX IV and LC3B in kidney lysates. (H) EM image of mitophagosomes in renal tubular epithelial cells of cisplatin-treated mice. (I-M) Immunoblot analysis and quantification of BNIP3, VDAC, COX IV and LC3B in HK-2 cell lysates. (N) Representative images of MitoTracker and LysoTracker with Hoechst to show the formation of mitophagosomes in HK-2 cells. Data are presented as the mean ± SEM (n=5 *in vivo*; n=3 *in vitro*). *p<0.05, **p<0.01 and ***p<0.001.

PINK1-PARK2-mediated mitophagy in cisplatin-induced acute kidney injury has been previously described 19. We verified these data and detected BNIP3-mediated mitophagy in cisplatin-treated mice. Cisplatin treatment upregulated the expression levels of BNIP3, PINK1, PARK2 and LC3B II but downregulated COX IV (mitochondria inner membrane marker) and VDAC (mitochondria outer membrane protein) in the renal cortex (Figure 2A-2G). TEM analysis revealed the formation of mitophagosomes in the renal tubular epithelial cells of cisplatin-treated mice (Figure 2H). In an* in vitro* study, immunoblot analysis also showed an increase in BNIP3 and LC3B II but a decrease in COX IV and VDAC (Figure 2I-2M). MitoTracker and LysoTracker probes demonstrated that cisplatin treatment promoted mitophagolysosome formation (Figure 2N). These data indicated that mitophagy is increased both *in vivo* and* in vitro* in cisplatin-induced acute kidney injury.

### 3. *Bnip3* deficiency aggravates cisplatin-induced acute kidney injury

**Fig 3:**
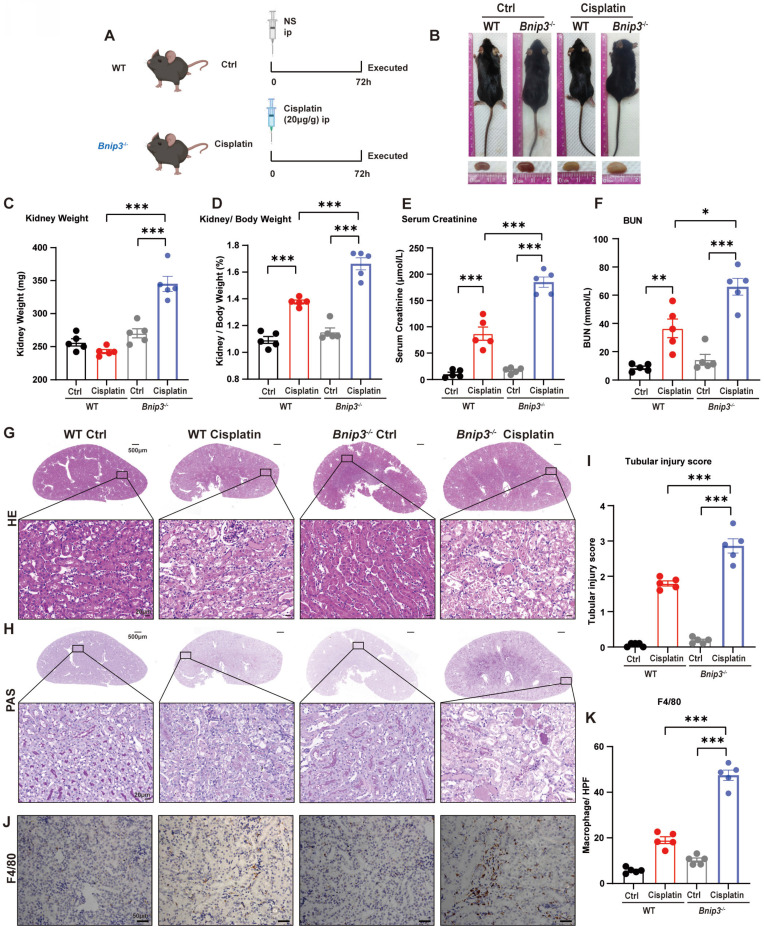
***Bnip3* deficiency aggravates cisplatin-induced acute kidney injury.** (A) Schematic representation of cisplatin-induced acute kidney injury in WT and *Bnip3* knockout mice. (B) Morphology of mice and kidneys before sacrifice. (C) Kidney weight and (D) kidney/body weight ratio at the prefusion time. (E, F) Renal function was evaluated by serum creatinine and BUN levels. (G-I) Representative histology and renal tubular injury score in the renal cortex according to HE and PAS staining. (J-K) Macrophage infiltration was measured by F4/80 immunobiological staining. Data are presented as the mean ± SEM (n=5). *p<0.05, **p<0.01 and ***p<0.001.

To clarify the function of BNIP3 and BNIP3-mediated mitophagy in cisplatin-induced acute kidney injury, we intraperitoneally injected cisplatin into* Bnip3* knockout mice (Figure 3A). In WT mice, cisplatin injection caused a significant reduction in body weight but no difference in kidney weight. Compared to WT cisplatin-treated mice, however, *Bnip3* knockout cisplatin-treated mice showed no difference in body weight but had larger kidneys (Figure 3B, 3C; Supplemental Figure 2A, 2B). The kidney/body weight ratio was increased due to relatively smaller body size after cisplatin injection in WT mice, and *Bnip3* knockout resulted in a larger kidney size compared to the WT cisplatin-treated group (Figure 3D). The serum creatinine and BUN levels suggested more serious renal damage in *Bnip3* knockout cisplatin-treated mice than in WT cisplatin-treated mice (Figure 3E, 3F). The tubular injury marker, kidney injury molecule 1 (KIM1), was also increased in* Bnip3* knockout cisplatin-treated mice compared to the WT cisplatin-treated group (Supplemental Figure 2C). HE and PAS staining showed severe epithelial flattening, exfoliation of epithelial cells, tubular basement membrane nakedness and cast formation in Bnip3 knockout cisplatin-treated mouse kidneys as well as higher tubular injury score than the WT cisplatin-treated group (Figure 3G-I). Inflammation in the kidney was evaluated by immunohistochemical staining of F4/80, which showed more macrophage infiltration in *Bnip3* knockout cisplatin-treated mice than in WT cisplatin-treated mice (Figure 3J, 3K).

### 4. Cisplatin-induced ROS, lipid peroxidation and RTEC ferroptosis are increased in *Bnip3* knockout kidneys

**Fig 4:**
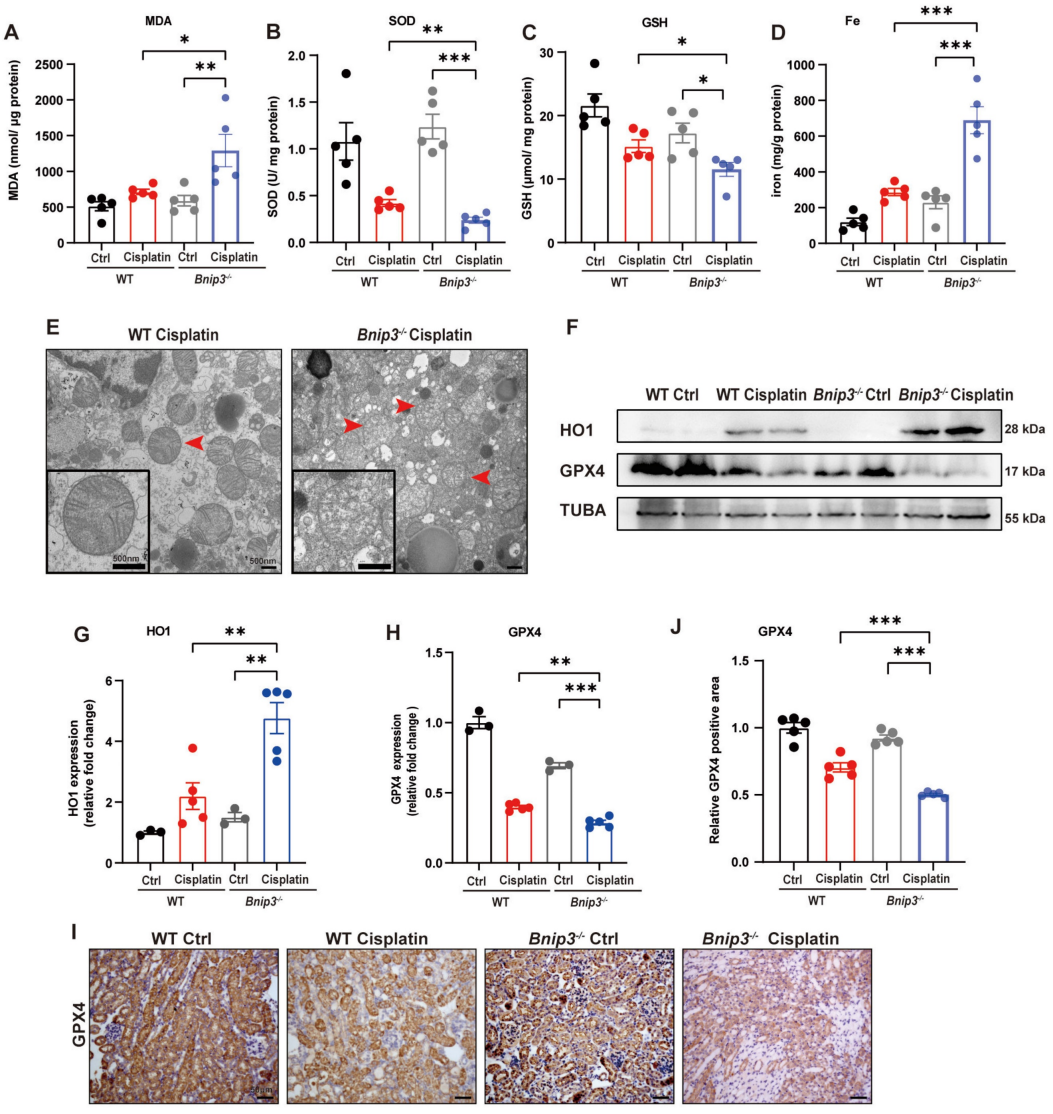
** Cisplatin-induced ROS, lipid peroxidation and RTEC ferroptosis are increased in *Bnip3* knockout kidneys.** (A, B) ROS were evaluated by MDA and SOD levels in WT and *Bnip3* knockout mice intraperitoneally injected with cisplatin. (C, D) Glutathione and iron levels in renal homogenate. (E) Representative EM image of mitochondrial injury in renal tubular epithelial cells of cisplatin-treated WT and *Bnip3* knockout mice. (F-H) Immunoblot analysis and quantification of HO1 and GPX4 in kidney lysates. (I, J) Immunohistochemical staining of GPX4. Data are presented as the mean ± SEM (n=5). *p<0.05, **p<0.01 and ***p<0.001.

The ROS level was measured by MDA and SOD levels. The data suggested higher MDA level and lower SOD level in the kidneys of *Bnip3* knockout cisplatin-treated mice compared to WT cisplatin-treated mice (Figure 4A, 4B). The GSH level revealed that *Bnip3* knockout further aggravated the reduction in glutathione caused by cisplatin (Figure 4C). Kidney iron levels showed that cisplatin-induced iron release was aggravated in *Bnip3* knockout mice compared to WT mice (Figure 4D). TEM analysis showed that mitochondrial injury was increased in *Bnip3* knockout cisplatin-treated mice, which was characterized by increased vacuolization in mitochondria and rupturing of mitochondrial cristae (Figure 4E). Immunoblot analysis and quantification of HO1 and GPX4 revealed that cisplatin-induced ferroptosis was exacerbated in *Bnip3* knockout kidneys (Figure 4F-4H), which was confirmed by immunohistochemical staining of GPX4 (Figure 4I, 4J). These data indicated that inhibition of BNIP3-mediated mitophagy aggravates cisplatin-induced ferroptosis in renal tubular epithelial cells.

### 5. Silencing *BNIP3* upregulates ROS and cell death, which are rescued by ferrstatin-1

**Fig 5:**
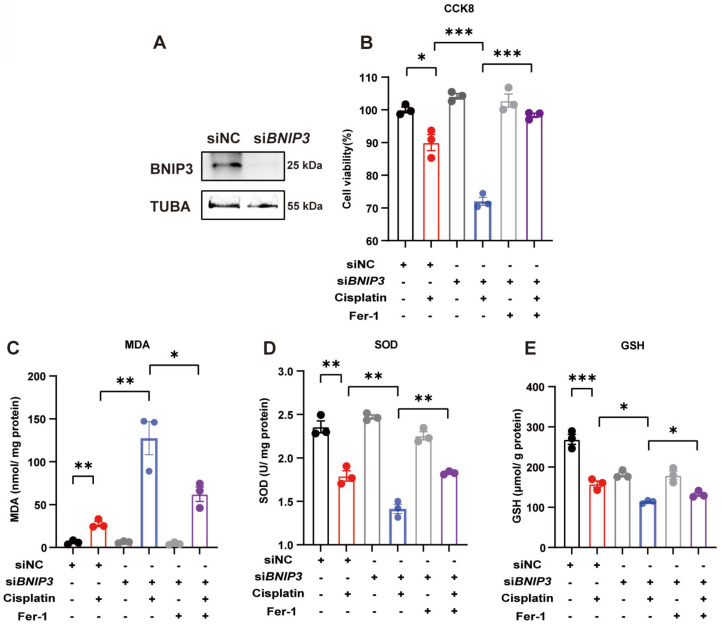
** Silencing* BNIP3* upregulates ROS and cell death, which are rescued by ferrstatin-1.** *BNIP3* siRNA was transfected into HK-2 cells to knockdown the *BNIP3* gene. (A) Representative image of BNIP3 after siRNA transfection. (B) CCK-8 assay of cisplatin-treated *BNIP3* knockdown HK-2 cells with or without ferrstatin-1, an antioxidant ferroptosis inhibitor. (C-E) MDA, SOD and GSH levels in cell homogenates were used to determine ROS and glutathione levels in HK-2 cells. n=3. *p<0.05, **p<0.01 and ***p<0.001.

We used siRNA to knockdown BNIP3 levels and evaluated cisplatin-induced ROS and cell death levels in HK-2 cells (Figure 5A). The CCK8 assay demonstrated that silencing BNIP3 aggravated cisplatin-induced cell death, but these effects were rescued by ferrstatin-1, a synthetic antioxidative ferroptosis inhibitor (Figure 5B). The MDA level was increased by BNIP3 silencing in the absence of ferrstatin-1 but was reduced in the ferrstatin-1 pretreatment group (Figure 5C). Cisplatin reduced the SOD and GSH levels in HK-2 cells, whereas ferrstatin-1 rescued the SOD and GSH levels (Figure 5D, 5E). These data demonstrated that cisplatin induces ROS and cell death in HK-2 cells, which are aggravated by inhibition of BNIP3-mediated mitophagy, and that ferrstatin-1 rescues ROS and injury caused by BNIP3 knockdown.

### 6. Silencing *BNIP3* exacerbates cisplatin-induced lipid peroxidation and ferroptosis

**Fig 6:**
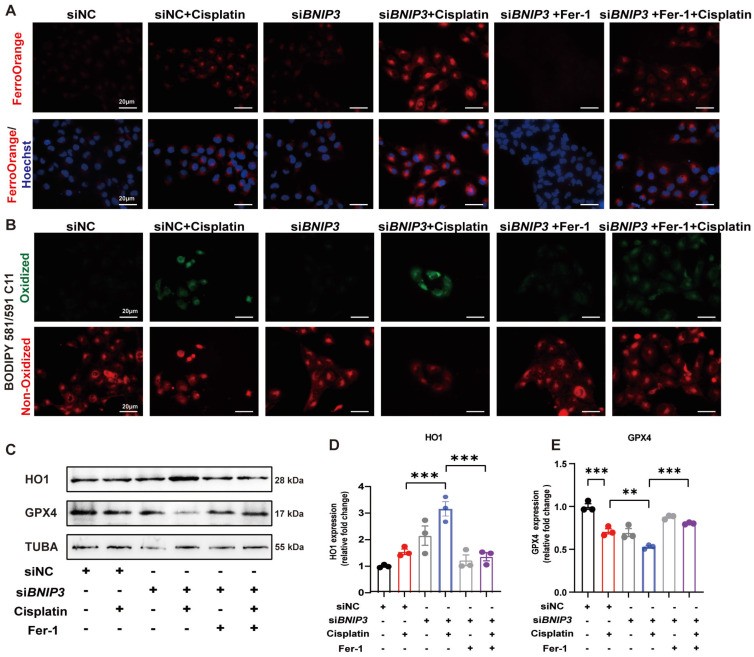
** Silencing* BNIP3* exacerbates cisplatin-induced lipid peroxidation and ferroptosis.** (A-B) FerroOrange and BODIPY 581/591 C11 probes showed Fe^2+^ and lipid peroxidation levels in cisplatin-treated *BNIP3* knockdown HK-2 cells with or without ferrstatin-1. (C-E) Immunoblot analysis and quantification of HO1 and GPX4 in HK-2 cell lysates. n=3. *p<0.05, **p<0.01 and ***p<0.001.

To detect iron metabolism and lipid peroxidation, FerroOrange and BODIPY 581/591 C11 probes were added after cisplatin incubation. FerroOrange staining showed that cisplatin upregulated Fe^2+^ in the cytoplasm, which was further increased by silencing *BNIP3* in HK-2 cells (Figure 6A). The BODIPY 581/591 C11 probe revealed that oxidized lipids were increased in the cisplatin group and were aggravated by silencing *BNIP3* (Figure 6B). Both Fe^2+^ release and lipid peroxidation caused by inhibition of BNIP3-mediated mitophagy were rescued by ferrstatin-1 (Figure 6A, 6B). Immunoblot analysis and quantification of HO1 and GPX4 were used to evaluate ferroptosis* in vitro*. The results indicated that cisplatin upregulated HK-2 cell ferroptosis and that *BNIP3* silencing exacerbated cisplatin-induced ferroptosis, which was reversed by ferrstatin-1 (Figure 6C-6E). These data demonstrated that BNIP3-mediated mitophagy limits the aggravation of cisplatin-induced ferroptosis through the ROS/HO1/GPX4 axis.

### 7. *Pink1* deficiency aggravates cisplatin-induced RTEC ferroptosis *in vivo*

**Fig 7:**
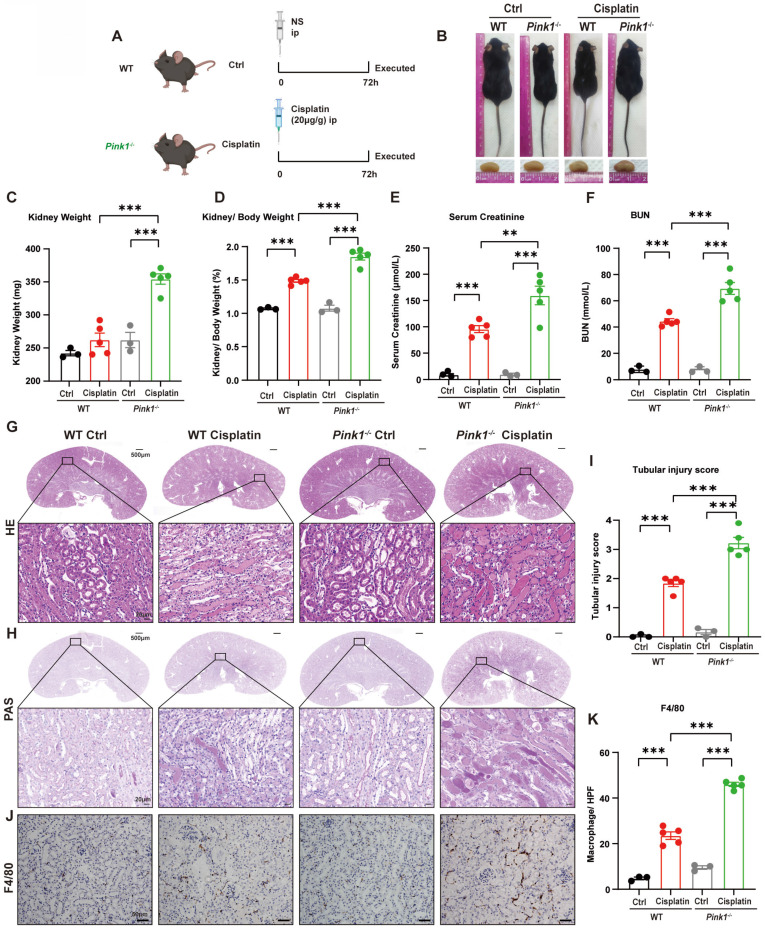
***Pink1* deficiency aggravates cisplatin-induced acute kidney injury.** (A) Schematic representation of cisplatin-induced acute kidney injury in WT and *Pink1* knockout mice. (B) Morphology of mice and kidneys before sacrifice. (C) Kidney weight and (D) kidney/body weight ratio at the prefusion time. (E, F) Renal function was evaluated by serum creatinine and BUN levels. (G-I) Representative histology and renal tubular injury score in the renal cortex according to HE and PAS staining. (J-K) Macrophage infiltration was measured by F4/80 immunobiological staining. Data are presented as the mean ± SEM (n=3-5). **p<0.01 and ***p<0.001.

Because we demonstrated that BNIP3-mediated mitophagy protected against cisplatin-induced ferroptosis, we further investigated the role of the PARK2-dependent mitophagy pathway in ferroptosis by intraperitoneally injecting cisplatin into both *Pink1* knockout mice and *Park2* knockout mice (Figure 7A, 9A). The body size did not significantly differ between *Pink1* knockout cisplatin-treated mice and WT cisplatin-treated mice (Figure 7B), which was confirmed by the body weights (Supplemental Figure 3A, 3B). The kidney weight and kidney/body weight ratio showed larger kidneys as well as increased serum creatinine, BUN and KIM1 levels in *Pink1* knockout cisplatin-treated mice, which indicated that *Pink1* knockout aggravated kidney injury (Figure 7D-7F; Supplemental Figure 3C). HE and PAS staining indicated increased cast formation, exfoliation of epithelial cells and tubular basement membrane nicks as well as higher tubular injury scores in *Pink1*-knockout cisplatin-treated kidneys than in WT cisplatin-treated kidneys (Figure 7G-7I). F4/80 staining showed severe inflammatory infiltration in *Pink1* knockout cisplatin-treated mice, which also demonstrated that *Pink1* knockout aggravated cisplatin-induced kidney injury.

**Fig 8:**
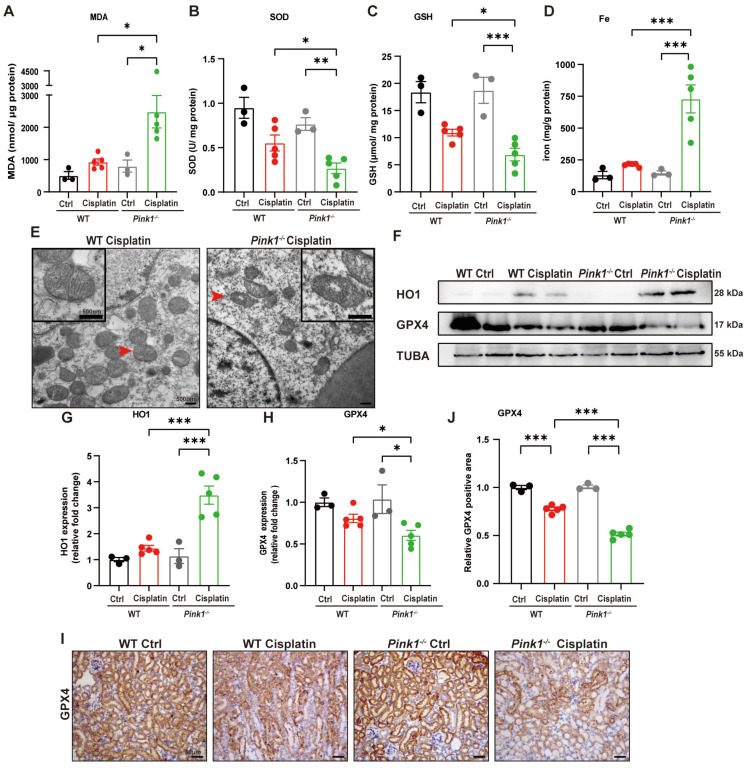
***Pink1* deficiency promotes cisplatin-induced ROS, lipid peroxidation and RTEC ferroptosis.** (A, B) ROS were evaluated by MDA and SOD levels in WT and *Pink1* knockout mice intraperitoneally injected with cisplatin. (C, D) GSH and iron levels in renal homogenate. (E) Representative EM image of mitochondrial injury in renal tubular epithelial cells of WT cisplatin-treated and *Pink1* knockout cisplatin-treated mice. (F-H) Immunoblot analysis and quantification of HO1 and GPX4 in kidney lysates. (I, J) Immunohistochemical staining of GPX4. Data are presented as the mean ± SEM (n=3-5). *p<0.05, **p<0.01 and ***p<0.001.

To further verify the involvement of PINK1 in ferroptosis, we evaluated SOD, MDA, GSH and iron levels (Figure 8A-8D). The results showed higher MDA levels and lower SOD and GSH levels as well as increased iron release in *Pink1* knockout cisplatin-treated mice compared to the WT cisplatin-treated group. Mitochondrial atrophy and vacuole formation were observed in *Pink1* knockout cisplatin-treated mice (Figure 8E). The upregulation of HO1 and downregulation of GPX4 indicated that cisplatin-induced ferroptosis was increased in the *Pink1* knockout group (Figure 8F-8H). Immunohistochemistry indicated less GPX4-positive staining in *Pink1* knockout cisplatin-treated kidneys than in WT cisplatin-treated kidneys (Figure 8I, 8J). These data demonstrated that inhibition of *Pink1 in vivo* aggravates cisplatin-induced ROS, ferroptosis and kidney injury.

### 8. *Park2* deficiency aggravates cisplatin-induced RTEC ferroptosis *in vivo*

**Fig 9:**
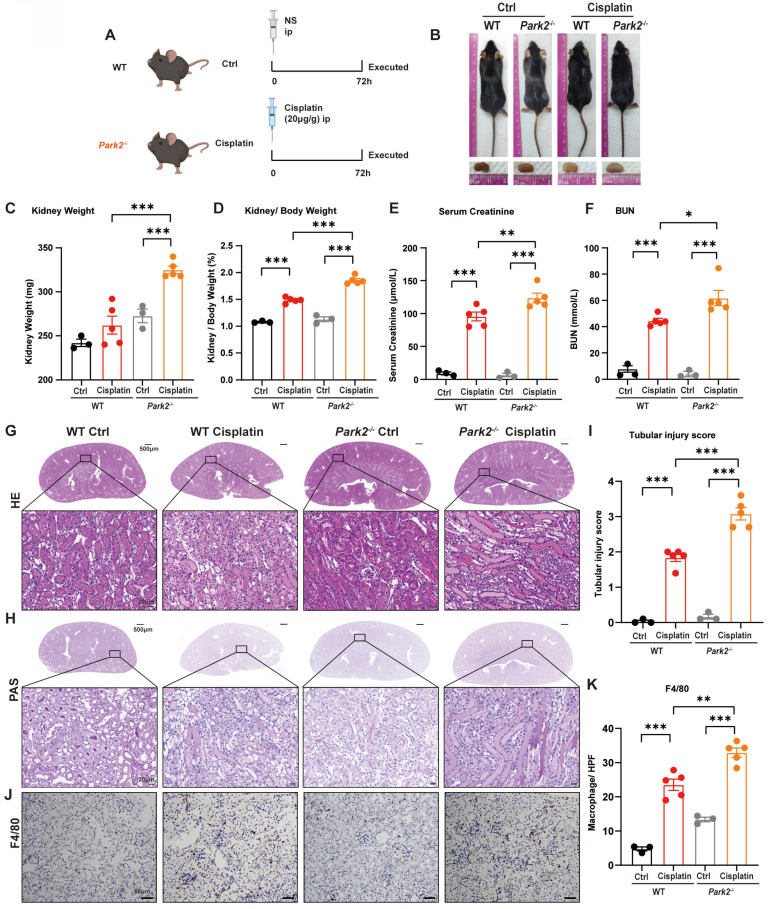
***Park2* deficiency aggravates cisplatin-induced acute kidney injury.** (A) Schematic representation of cisplatin-induced acute kidney injury in WT and *Park2* knockout mice. (B) Morphology of mice and kidneys before sacrifice. (C) Kidney weight and (D) kidney/body weight ratio at the prefusion time. (E, F) Renal function was evaluated by serum creatinine and BUN levels. (G-I) Representative histology and renal tubular injury score in the renal cortex according to HE and PAS staining. (J-K) Macrophage infiltration was measured by F4/80 immunobiological staining. Data are presented as the mean ± SEM (n=3-5). *p<0.05, **p<0.01 and ***p<0.001.

We intraperitoneally injected cisplatin into *Park2* knockout mice to elucidate the role of PARK2-mediated mitophagy and ferroptosis (Figure 9A). The body weight and body weight change were not significantly different in *Park2* knockout mice and WT mice treated with cisplatin (Figure 9B; Supplemental Figure 4A, 4B). The kidney appearance, kidney weight and kidney/body weight ratio showed that *Park2* knockout cisplatin-treated kidneys were larger than those of the WT cisplatin-treated group (Figure 9B-9D). The serum creatinine, BUN and KIM1 levels were also increased in the *Park2* knockout group (Figure 9E, 9F; Supplemental Figure 4C). HE and PAS staining showed increased cast formation and intraepithelial vacuolar degeneration as well as a higher tubular injury score in *Park2* knockout cisplatin-treated mice compared to WT cisplatin-treated mice (Figure 9G-9I). Immunohistochemical staining of F4/80 demonstrated more inflammatory infiltration in *Park2* knockout kidneys (Figure 9J, 9K). These data indicated that *Park2* knockout aggravates cisplatin-induced kidney injury.

**Fig 10:**
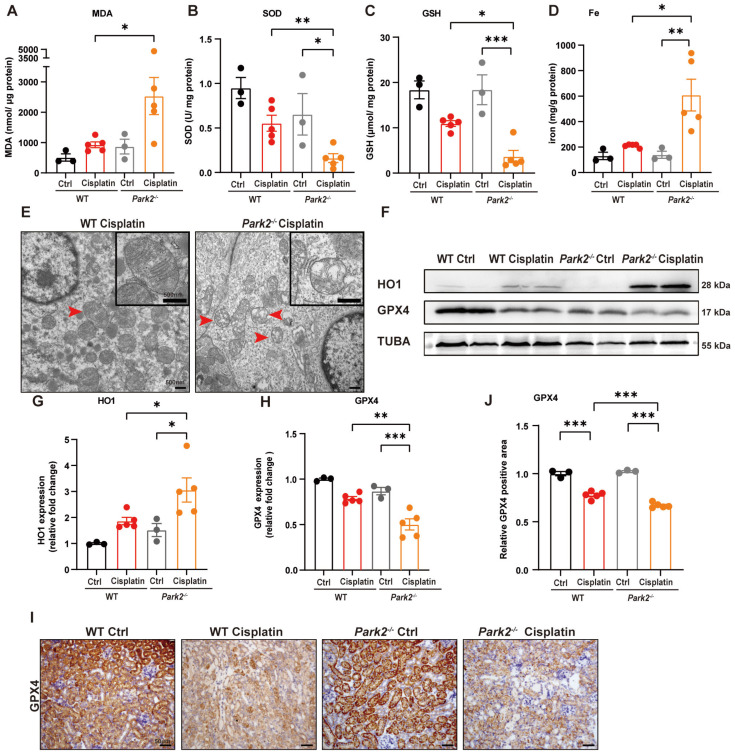
***Park2* deficiency promotes cisplatin-induced ROS, lipid peroxidation and RTEC ferroptosis.** (A, B) ROS were evaluated by MDA and SOD levels in WT and *Park2* knockout mice intraperitoneally injected with cisplatin. (C, D) GSH and iron levels in renal homogenate. (E) Representative EM image of mitochondrial injury in renal tubular epithelial cells of cisplatin-treated WT and *Park2* knockout mice. (F-H) Immunoblot analysis and quantification of HO1 and GPX4 in kidney lysates. (I, J) Immunohistochemical staining of GPX4. Data are presented as the mean ± SEM (n=3-5). *p<0.05, **p<0.01 and ***p<0.001.

The role of Park2-mediated mitophagy and ferroptosis was evaluated by ROS, ferroptosis-related mitochondrial damage and key protein levels. *Park2* knockout upregulated MDA and iron levels but downregulated SOD and GSH levels, which suggested that PARK2 deficiency resulted in an increase in ROS, loss of glutathione and iron release (Figure 10A-10D). TEM analysis showed increased severe mitochondrial outer membrane rupture and vacuolization in mitochondria in *Park2* knockout renal tubular epithelial cells (Figure 10E). Immunoblot analysis and quantification of HO1 and GPX4 indicated that ferroptosis was increased in *Park2* knockout cisplatin-treated mice (Figure 10F-10H). Immunohistochemical staining of GPX4 also showed less positive staining in the tubules of *Park2* knockout cisplatin-treated mice compared to WT cisplatin-treated mice (Figure 10I, 10J). Taken together, these data demonstrated that PINK1-PARK2-mediated mitophagy protects against cisplatin-induced acute kidney injury by inhibiting renal tubular epithelial cell ferroptosis.

## Discussion

**Fig 11:**
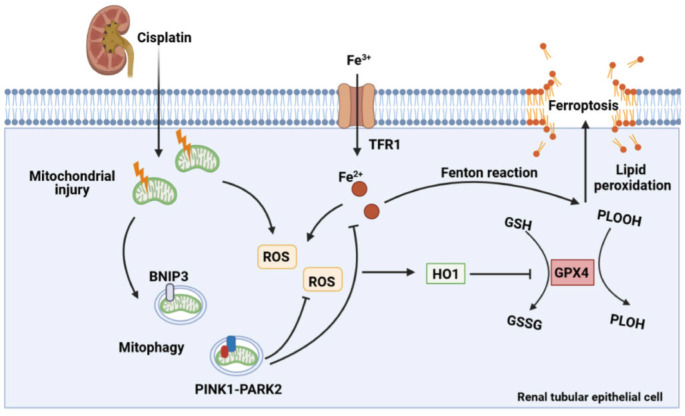
** Schematic representation of mitophagy, ROS, iron metabolism and ferroptosis in cisplatin-induced acute kidney injury.** Cisplatin causes mitochondrial injury, which releases ROS to aggravate injury and activate the mitochondrial protective mechanism of mitophagy to limit injury exacerbation. Both BNIP3- and PINK1-/PARK2-mediated mitophagy reduce excessive ROS release and HO1 expression and then rescues GPX4 downregulation, which decreases lipid peroxidation and ferroptosis. Moreover, mitophagy inhibits Fe^2+^ production and the Fenton reaction, resulting in an increase in GPX4 and a reduction in ferroptosis. Thus, mitophagy protects against cisplatin-induced acute kidney injury by inhibiting ferroptosis in renal tubular epithelial cells.

In the present study, the effect of mitophagy on cisplatin-induced ferroptosis was examined using *Bnip3* knockout, *Pink1* knockout and *Park2* knockout mice. The present results showed that cisplatin induced mitochondrial injury, ROS release, intracellular iron accumulation, lipid peroxidation, HO1 signaling upregulation, and GPX4 loss, which resulted in renal tubular epithelial cell ferroptosis and acute kidney injury. Additionally, inhibiting BNIP3-mediated or PINK1-PARK2-mediated mitophagy increased oxidative stress and lipid peroxidation, which aggravated ferroptosis. Ferrstatin-1, a synthetic antioxidative ferroptosis inhibitor, rescued iron accumulation, lipid peroxidation and ferroptosis caused by inhibition of mitophagy. Importantly, *Bnip3* knockout, *Pink1* knockout and *Park2* knockout mice showed more severe kidney injury than WT mice, suggesting the important roles of mitophagy and mitochondrial quality control in cisplatin-induced acute kidney injury (Figure 11). Notably, we confirmed a new mechanism by which mitophagy protects against cisplatin-induced acute kidney injury by decreasing renal tubular epithelial cell ferroptosis, suggesting that mitophagy and ferroptosis are new therapeutic targets for the prevention and treatment of acute kidney injury.

Autophagy is considered a double-edged sword 35, 36. In kidney research, most studies have supported the protective role of autophagy in kidney diseases 16, 37, 38. Other studies have also shown that persistent activation of autophagy in kidney tubular cells promotes renal interstitial fibrosis 39, 40. Mitophagy plays a protective role in cell survival by degrading dysfunctional mitochondria and eliminating cytochrome c and excessive ROS released from damaged mitochondria 19, 41. In acute kidney injury research, Tang *et al.* 42, 43 showed the protective roles of the PINK1-PARK2 and BNIP3 pathways in ischemia reperfusion-induced acute kidney injury. Wang *et al.* 44 showed the protective roles of PINK1-PARK2 in septic acute kidney injury. Moreover, our laboratory has demonstrated the roles of PINK1-PARK2- and BNIP3-mediated mitophagy in contrast-induced acute kidney injury 17, 18. Several studies have shown that PINK1-PARK2-mediated mitophagy protects against cisplatin-induced acute kidney injury 19, 45, 46. The previous elucidated mechanisms have mostly focused on mitophagy eliminating damaged mitochondria and decreasing dynamin-related protein 1 (DRP1) as well as mitochondrial fission, cell necrosis and cell apoptosis. The present findings also demonstrated the protective role of PINK1-PARK2-mediated mitophagy (Figure 7, 9). However, few studies have emphasized the BNIP3-independent pathway. Zhou* et al.* 46 reported that BNIP3L protein is upregulated in cisplatin-induced acute kidney injury rats, and they suggested that the BNIP3/BNIP3L pathway may participate in cisplatin-induced mitophagy. Here, we intraperitoneally injected cisplatin into *Bnip3* knockout mice, which demonstrated that serum creatinine, pathological injury and inflammatory infiltration were exacerbated in cisplatin-treated Bnip3 knockout mice (Figure 3). Moreover, we demonstrated the protective function of BNIP3-mediated mitophagy *in vitro* by transfecting *BNIP3* siRNA into the HK-2 cell line (Figure 5). Further, the present study used *Pink1* knockout, *Park2* knockout and *Bnip3* knockout mice to confirm the protective role of both PINK1-PARK2-mediated and BNIP3-mediated mitophagy in cisplatin-induced acute kidney injury. Taken together, these data indicated that both PARK2-dependent and PARK2-independent mitophagy pathways protect against acute kidney injury. Thus, additional studies focusing on mitophagy regulation will help to improve the prognosis of acute kidney injury.

Ferroptosis is a type of programmed cell death driven by iron-dependent phospholipid peroxidation 22. Recent studies have demonstrated that ferroptosis is increased in acute kidney injury. Inducible *Gpx4* knockout mice directly aggravate lipid-oxidation-induced acute kidney injury and associated death 33. Melatonin treatment significantly alleviates ischemia reperfusion-induced ferroptosis and acute kidney injury 47. Folic acid induces ferroptosis, not necroptosis, in acute kidney injury 27, which can be rescued by quercetin through inactivation of transcription factor 3 28. Several studies have also demonstrated ferroptosis in cisplatin-induced acute kidney injury. Deng *et al.* 11 showed that cisplatin-induces ferroptosis through myo-inositol oxygenase regulated GSH and GPX4 activity as well as ferritinophagy 11. Fan *et al.* 48 indicated that hemopexin is a mediator of iron toxicity in the kidney, and deferoxamine alleviates cisplatin-induced acute kidney injury. Kim *et al.* 30 used genetic and pharmacological methods to demonstrate that farnesoid X receptor regulates the transcription of ferroptosis genes and reduces cisplatin-induced acute kidney injury. Additionally, the vitamin D receptor, ferrostatin-1 and small GTPase (Ras homolog enriched in brain) also decrease cisplatin-induced nephrotoxicity by ferroptosis 31, 49. The present results confirmed these previously findings. The present study also showed renal tubular epithelial cell ferroptosis *in vivo* and* in vitro* using models of cisplatin-induced acute kidney injury, and we demonstrated that the ROS/HO1/GPX4 axis contributes to ferroptosis (Figure 1). Moreover, ferrostatin-1, a synthetic antioxidant, inhibited cisplatin-induced ferroptosis by reducing ROS, Fe^2+^ release and lipid peroxidation (Figure 5, 6). Taken together, these findings indicated that ferroptosis aggravates acute kidney injury, suggesting that ferroptosis inhibitors may be therapeutic targets for clinical acute kidney injury treatment.

Recent published studies have focused on autophagy and ferroptosis. The mTOR signaling inhibition upregulates autophagy-mediated GPX4 degradation, thereby promoting ferroptosis of bladder cancer cells 50. The AMPK pathway phosphorylates BECN1 at Ser90/93/96, enhancing BECN1-SLC7A11 complex formation and subsequently inducing lipid peroxidation and ferroptosis 51. New ferroptosis-related autophagy includes ferritinophagy, lipophagy and clockophagy^52^, ^53^, which should be investigated in future kidney disease studies. The controversy between autophagic cell death and cell death accompanied by autophagy remains unclear 54. The results of the present study support the latter opinion. In the present study, cisplatin upregulated both ferroptosis and mitophagy, specifically autophagy (Figure 1,2), and genetic inhibition of BNIP3- or PINK1-PARK2-mediated mitophagy resulted in aggravation of ferroptosis in cisplatin-induced acute kidney injury (Figure 3, 5, 7,9). Thus, the present findings suggested that mitophagy is an upstream protective mechanism that prevents excessive ferroptotic cell death.

In the present study, we focused on mitophagy and ferroptosis in cisplatin-induced acute kidney injury. Few studies have investigated mitophagy and ferroptosis in the kidneys. Previous studies on other diseases have suggested that mitophagy-dependent ferroptosis contributes to cell death. Yu* et al.* 55 showed that inhibition of O-GlcNAcylation enhances mitophagy, releases labile iron and renders cells more sensitive to ferroptosis. Rademaker *et al.* 56 indicated that the mitophagy inhibitor, Mdivi1, decreases myoferlin-related ROS accumulation, lipid peroxidation and ferroptosis. Basit* et al.* 57 and Li *et al.* 58 also reported mitophagy-dependent ferroptosis in melanoma cells and Alzheimer's disease. The present study also found that cisplatin induced the upregulation of mitophagy, ROS production, lipid peroxidation and ferroptosis (Figure 1). However, previous studies have explained this phenomenon as mitophagy-dependent ferroptosis. Thus, we used genetic mitophagy gene (*Bnip3*, *Pink1* and *Park2*) knockout mice or knockdown cell lines to inhibit mitophagy combined with the cisplatin-induced acute kidney model *in vivo* and *in vitro* to further discuss the relationship between mitophagy and ferroptosis. The results showed that ferroptosis was aggravated and that ROS production, Fe^2+^ release and lipid peroxidation were increased during mitophagy inhibition (Figure 4, 6, 8, 10). Additionally, we showed that ferrostatin-1 pretreatment rescued the ROS/HO1/ferroptosis axis, which was upregulated by BNIP3 silencing (Figure 6). Importantly, we demonstrated that mitophagy protected against cisplatin-induced ferroptosis via the ROS/HO1/GPX4 pathway and proposed a new hypothesis of ferroptosis with mitophagy upregulation, which was different from the previously proposed mitophagy-related ferroptosis. Li *et al.* 59 demonstrated that hypoxia inducible factor (HIF) promotes mitophagy and modulates redox homeostasis, which may protect against ferroptosis in acute kidney injury. Additional studies on hypoxia, mitophagy and ferroptosis are currently ongoing.

The renal volume of acute kidney injury is significantly larger than that of healthy individuals. In the present study, we used kidney/body weight to evaluate the renal volume. As shown in Figure 3, 7 and 9, the renal volume of the WT cisplatin-treated group was larger than that of the WT ctrl group, and the *Bnip3* knockout, *Pink1* knockout and *Park2* knockout groups had higher kidney/body weight and creatinine levels than the WT cisplatin-treated group, suggesting that the renal volume was correlated with kidney injury. Interestingly, although the kidney/body weight ratio had a similar trend in the above two comparison groups, the detailed comparisons showed many differences. Compared to the WT ctrl group, WT cisplatin-treated mice had lower body weights due to cisplatin injection, but the kidney weights were not significantly different. Nevertheless, the body weight of *Bnip3*/*Pink1*/*Park2* knockout cisplatin-treated mice was not different from that of WT cisplatin-treated mice. The kidney weight of *Bnip3*/*Pink1*/*Park2* knockout cisplatin-treated mice was much higher than that of WT cisplatin-treated mice. Accordingly, cisplatin injection reduced body weight 60 without causing kidney weight loss in 72 hours, which caused cisplatin-induced acute kidney injury. However, the aggravation of cisplatin-induced acute kidney injury in *Bnip3*/*Pink1*/*Park2* knockout mice was not attributed to only the pharmacological action of cisplatin, which indicated that mitophagy may have an effect on renal volume. This hypothesis will be further investigated in the future.

The present study had some limitations. The *Bnip3*^-/-^, *Pink1*^-/-^ and *Park2*^-/-^ mice were all global knockout mice. Tubular-specific knockout mice will better show the protective role of mitophagy in cisplatin-induced ferroptosis and kidney injury.

In summary, the present study demonstrated that mitophagy alleviates cisplatin-induced tubular cell ferroptosis through the ROS/HO-1/GPX4 axis. Moreover, renal tubular epithelial cell ferroptosis exacerbates cisplatin-induced acute kidney injury. Mitophagy is activated to reduce excessive ferroptosis and kidney injury, while inhibition of BNIP3- or PINK1-PARK2-mediated mitophagy aggravates ROS release, lipid peroxidation, cell ferroptosis and cisplatin nephrotoxicity. The present findings indicated that ferroptosis inhibitors and mitophagy agonists are potential therapeutic targets for the clinical prevention and treatment of chemotherapeutic drug-induced acute kidney injury.

## Methods

### Animals and cisplatin-induced acute kidney injury model

*Bnip3* knockout mice were constructed at Shanghai Model Organisms Center, Inc. as previously described 61 and verified in our previous study 18. *Pink1* knockout mice (017946) and *Park2* knockout mice (006582) with a C56BL/6 genetic background were purchased from Jackson Laboratory and described in our previously published report^17^. All animal experiments were approved by the Animal Care Committee of Renji Hospital, School of Medicine, Shanghai Jiao Tong University. Male mice, aged 6-8 weeks and weighing 20-25 g, were administered cisplatin (20 mg/kg, Jiangsu Hansoh, H20040813) or normal saline through intraperitoneal injection (ip)^30^, ^32^. The mice were sacrificed after 72 hours to harvest serum and kidneys for subsequent analyses.

### *In vitro* cisplatin treatment

The human renal proximal tubular cell line (HK-2 cell line) was obtained from the American Type Culture Collection (ATCC®, CRL-2190) and cultured in Dulbecco's modified Eagle's medium (DMEM)/F-12 (Thermo Fisher Scientific, 11330057, New York, USA) supplemented with 1% penicillin‒streptomycin (Thermo Fisher Scientific, 10378016) and 10% fetal bovine serum (Thermo Fisher Scientific, 10099158). HK-2 cells were transfected with 50 nm *BNIP3* siRNA for 8 hours by Lipofectamine™ 3000 Transfection Reagent (Thermo Fisher Scientific, L3000150). Two hours before cisplatin administration, HK-2 cells were pretreated with ferrostatin-1 (10 μM, MCE, HY-100579) 62 followed by treatment with cisplatin (20 μM) for 24 hours, and cells were then harvested 63.

The following small interfering RNA (siRNA) sequence was used, which was confirmed in our previous work 18: *BNIP3* siRNA 5′-CGUUCCAGCCUCGGUUUCUAUUUAU-3′. Experiments were performed in triplicate.

### Renal function, histopathology and immunohistochemical staining

Serum creatinine and BUN were used to evaluate the renal function of mice. The serum creatinine (Nanjing Jiancheng Bioengineering Institute, C011-2-1) and BUN (Nanjing Jiancheng Bioengineering Institute, C013-2-1) levels were measured according to the manufacturer's instructions. HE staining and PAS staining of kidneys were performed as previously described 64, 65. The tubular injury score was based on the percentage of damaged tubules as follows: grade 0, no damage; grade 1, injured tubules less than 25%; grade 2, 25%<injured tubules≤50%; grade 3, 50%<injured tubules≤75%; and grade 4, injured tubules more than 75%.

For immunohistochemical analyses, 4-μm thick paraffin-embedded kidney sections were deparaffinized using dimethylbenzene. After ethylenediaminetetraacetic acid retrieval, the kidney sections were incubated with anti-F4/80 (1:100, Cell Signal Technology, 70076, MA, USA), a marker for macrophage infiltration, and anti-GPX4 (1:100, Abcam ab125066, Cambridge, UK), a marker for ferroptosis, at 4 °C overnight. The images were captured using ZEISS and Axio Vert A1, and they were quantified by ImageJ (ImageJ bundled with 64-bit Java 8).

To label the mitophagosomes, we used MitoTracker Green (50 nM, ThermoFisher Scientific, M7514), LysoTracker Red (50 nM, Beyotime, C1046) and Hoechst (5 μg/mL) to label mitochondria, lysosomes and nuclei, respectively, following the manufacturer's instructions. The images were captured using a ZEISS Axio Vert A1 microscope.

### Transmission electron microscopy

The fresh kidney was cut into 1^ mm3^ pieces and prefixed in 2% glutaraldehyde. The fixation, dehydration, embedding, polymerization, and lead citrate staining were performed by the Core Facility of Basic Medical Sciences, Shanghai Jiao Tong University of Medicine, as described previously 17. An H-7650 transmission electron microscope (Hitachi, H-7650) was used to detect 70-nm thick sections.

### Cell viability analysis

A CCK-8 kit (Dojindo, CK04) was used to analyze the viability of HK-2 cells. Briefly, after treatment with cisplatin, 10 μl of CCK8 solution was added to cell culture followed by incubation at 37 °C for 4 hours. BioTak CytationTM^3^ was used to measure the absorbance at 450 nm every 30 minutes.

### Immunoblot analysis

The proteins were subjected to 10-12% gel electrophoresis as described previously 66. The membranes were incubated with the following primary antibodies (1:1000 dilution) at 4 °C overnight: TFRC (Cell Signal Technology, 46222, MA, USA), HO1 (Cell Signal Technology, 43966, MA, USA), GPX4 (Abcam, ab125066, Cambridge, UK), BNIP3 (Santa Cruz Biotechnology, sc-56167, TX, USA), PINK1 (Novas Biologicals, BC100-494, CO, USA), PARK2 (Cell Signal Technology, 2132, MA, USA), VDAC (Abcam, ab14734, Cambridge, UK), COX IV (Cell Signal Technology, 4844, MA, USA), LC3B (Sigma‒Aldrich, L7543, MO, USA), KIM1 (R&D, AF1817, MN, USA) and TUBA (Beyotime, AF0001, Shanghai, China).

### ROS and ferroptosis analyses

ROS activity was detected by MDA (Beyotime, S0131S) and SOD (Nanjing Jiancheng Bioengineering Institute, A001-3-2) levels according to the manufacturer's instructions. The MDA and SOD levels were adjusted by protein concentration.

A GSH kit (Nanjing Jiancheng Bioengineering Institute, A006-2-1) was used to evaluate glutathione levels. The FerroOrange (1 μM, Dojindo, F374) probe was added to the cell culture to label Fe^2+^, and the BODIPY™ 581/591 C11 (10 μM, ThermoFisher Scientific, M7514) probe distinguished oxidized and nonoxidized lipids 30.

### Statistical analysis

GraphPad Prism 8 (GraphPad) was used for all statistical analyses. The qualitative data are presented as the mean± standard error (SEM). Student's t test was used to compare the differences between two groups, and one-way analysis of variance and Tukey's post hoc test were used to compare differences between more than two groups. A P value less than 0.05 was considered to indicate a significant difference.

## Supplementary Material

Supplementary figures.

## Abbreviations

BNIP3: BCL2/adenovirus E1B interacting protein 3
Ctrl: control
DAPI: 4′,6-diamidino-2-phenylindole dihydrochloride
GPX4: glutathione peroxidase 4
GSH: glutathione
HE: hematoxylin and eosin
HO1: heme oxygenase 1
KIM1: kidney injury molecule 1
MAP1LC3B/LC3B: microtubule-associated protein 1 light chain 3 beta
MDA: malondialdehyde
NS: normal saline
PARK2/Parkin: parkin RBR E3 ubiquitin protein ligase
PINK1: PTEN induced putative kinase 1
ROS: reactive oxygen species
RTEC: renal tubular epithelial cell
SEM: standard error of the mean
siRNA: small interfering RNA
SOD: superoxide dismutase
TEM: transmission electron microscopy
TFRC: transferrin receptor 1
TUBA/α-tubulin: tubulin, alpha
TUNEL: terminal deoxynucleotidyl transferase-mediated dUTP nick end labeling
VDAC: voltage-dependent anion channel
WT: wild-type

## References

[1] Perazella MA, Rosner MH (2022). Drug-Induced Acute Kidney Injury. Clin J Am Soc Nephrol.

[2] Hoste EA, Bagshaw SM, Bellomo R (2015). Epidemiology of acute kidney injury in critically ill patients: the multinational AKI-EPI study. Intensive Care Med.

[3] Liu C, Yan S, Wang Y (2021). Drug-Induced Hospital-Acquired Acute Kidney Injury in China: A Multicenter Cross-Sectional Survey. Kidney Dis (Basel).

[4] He J, Su C, Liang W (2021). Icotinib versus chemotherapy as adjuvant treatment for stage II-IIIA EGFR-mutant non-small-cell lung cancer (EVIDENCE): a randomised, open-label, phase 3 trial. Lancet Respir Med.

[5] Siefker-Radtke AO, Necchi A, Park SH (2022). Efficacy and safety of erdafitinib in patients with locally advanced or metastatic urothelial carcinoma: long-term follow-up of a phase 2 study. Lancet Oncol.

[6] Yamada Y, Boku N, Mizusawa J (2019). Docetaxel plus cisplatin and S-1 versus cisplatin and S-1 in patients with advanced gastric cancer (JCOG1013): an open-label, phase 3, randomised controlled trial. Lancet Gastroenterol Hepatol.

[7] Hu X, Ma Z, Wen L (2021). Autophagy in Cisplatin Nephrotoxicity during Cancer Therapy. Cancers (Basel).

[8] Zsengeller ZK, Ellezian L, Brown D (2012). Cisplatin nephrotoxicity involves mitochondrial injury with impaired tubular mitochondrial enzyme activity. J Histochem Cytochem.

[9] Yan M, Shu S, Guo C (2018). Endoplasmic reticulum stress in ischemic and nephrotoxic acute kidney injury. Ann Med.

[10] Takai N, Abe K, Tonomura M (2015). Imaging of reactive oxygen species using [(3)H]hydromethidine in mice with cisplatin-induced nephrotoxicity. EJNMMI Res.

[11] Deng F, Sharma I, Dai Y (2019). Myo-inositol oxygenase expression profile modulates pathogenic ferroptosis in the renal proximal tubule. J Clin Invest.

[12] Klionsky DJ, Abdel-Aziz AK, Abdelfatah S Guidelines for the use and interpretation of assays for monitoring autophagy (4th edition) Autophagy. 2021; 17: 1-382.

[13] Shen ZF, Li L, Zhu XM (2022). Current opinions on mitophagy in fungi. Autophagy.

[14] Youle RJ, Narendra DP (2011). Mechanisms of mitophagy. Nat Rev Mol Cell Biol.

[15] Evans TD, Sergin I, Zhang X (2017). Target acquired: Selective autophagy in cardiometabolic disease. Sci Signal.

[16] Tang C, Livingston MJ, Liu Z (2020). Autophagy in kidney homeostasis and disease. Nat Rev Nephrol.

[17] Lin Q, Li S, Jiang N (2019). PINK1-parkin pathway of mitophagy protects against contrast-induced acute kidney injury via decreasing mitochondrial ROS and NLRP3 inflammasome activation. Redox Biol.

[18] Lin Q, Li S, Jiang N (2021). Inhibiting NLRP3 inflammasome attenuates apoptosis in contrast-induced acute kidney injury through the upregulation of HIF1A and BNIP3-mediated mitophagy. Autophagy.

[19] Wang Y, Tang C, Cai J (2018). PINK1/Parkin-mediated mitophagy is activated in cisplatin nephrotoxicity to protect against kidney injury. Cell Death Dis.

[20] Zhou RP, Chen Y, Wei X (2020). Novel insights into ferroptosis: Implications for age-related diseases. Theranostics.

[21] Wang D, Tang L, Zhang Y (2022). Regulatory pathways and drugs associated with ferroptosis in tumors. Cell Death Dis.

[22] Jiang X, Stockwell BR, Conrad M (2021). Ferroptosis: mechanisms, biology and role in disease. Nat Rev Mol Cell Biol.

[23] Deng HF, Yue LX, Wang NN (2020). Mitochondrial Iron Overload-Mediated Inhibition of Nrf2-HO-1/GPX4 Assisted ALI-Induced Nephrotoxicity. Front Pharmacol.

[24] Zhu L, Chen D, Zhu Y (2021). GPX4-Regulated Ferroptosis Mediates S100-Induced Experimental Autoimmune Hepatitis Associated with the Nrf2/HO-1 Signaling Pathway. Oxid Med Cell Longev.

[25] Liu J, Kuang F, Kroemer G (2020). Autophagy-Dependent Ferroptosis: Machinery and Regulation. Cell Chem Biol.

[26] Wang Y, Zhang M, Bi R (2022). ACSL4 deficiency confers protection against ferroptosis-mediated acute kidney injury. Redox Biol.

[27] Martin-Sanchez D, Ruiz-Andres O, Poveda J (2017). Ferroptosis, but Not Necroptosis, Is Important in Nephrotoxic Folic Acid-Induced AKI. J Am Soc Nephrol.

[28] Wang Y, Quan F, Cao Q (2021). Quercetin alleviates acute kidney injury by inhibiting ferroptosis. J Adv Res.

[29] Zhao Z, Wu J, Xu H (2020). XJB-5-131 inhibited ferroptosis in tubular epithelial cells after ischemia-reperfusion injury. Cell Death Dis.

[30] Kim DH, Choi HI, Park JS (2022). Farnesoid X receptor protects against cisplatin-induced acute kidney injury by regulating the transcription of ferroptosis-related genes. Redox Biol.

[31] Hu Z, Zhang H, Yi B (2020). VDR activation attenuate cisplatin induced AKI by inhibiting ferroptosis. Cell Death Dis.

[32] Portilla D, Li S, Nagothu KK (2006). Metabolomic study of cisplatin-induced nephrotoxicity. Kidney Int.

[33] Friedmann Angeli JP, Schneider M, Proneth B (2014). Inactivation of the ferroptosis regulator Gpx4 triggers acute renal failure in mice. Nat Cell Biol.

[34] Li Y, Feng D, Wang Z (2019). Ischemia-induced ACSL4 activation contributes to ferroptosis-mediated tissue injury in intestinal ischemia/reperfusion. Cell Death Differ.

[35] Choi Y, Bowman JW, Jung JU (2018). Autophagy during viral infection - a double-edged sword. Nat Rev Microbiol.

[36] Wu D, Wang H, Teng T (2018). Hydrogen sulfide and autophagy: A double edged sword. Pharmacol Res.

[37] Shi M, Maique J, Shepard S (2022). In vivo evidence for therapeutic applications of beclin 1 to promote recovery and inhibit fibrosis after acute kidney injury. Kidney Int.

[38] Li H, Peng X, Wang Y (2016). Atg5-mediated autophagy deficiency in proximal tubules promotes cell cycle G2/M arrest and renal fibrosis. Autophagy.

[39] Livingston MJ, Ding HF, Huang S (2016). Persistent activation of autophagy in kidney tubular cells promotes renal interstitial fibrosis during unilateral ureteral obstruction. Autophagy.

[40] Baisantry A, Bhayana S, Rong S (2016). Autophagy Induces Prosenescent Changes in Proximal Tubular S3 Segments. J Am Soc Nephrol.

[41] Tang C, Cai J, Yin XM (2021). Mitochondrial quality control in kidney injury and repair. Nat Rev Nephrol.

[42] Tang C, Han H, Yan M (2018). PINK1-PRKN/PARK2 pathway of mitophagy is activated to protect against renal ischemia-reperfusion injury. Autophagy.

[43] Tang C, Han H, Liu Z (2019). Activation of BNIP3-mediated mitophagy protects against renal ischemia-reperfusion injury. Cell Death Dis.

[44] Wang Y, Zhu J, Liu Z (2021). The PINK1/PARK2/optineurin pathway of mitophagy is activated for protection in septic acute kidney injury. Redox Biol.

[45] Ma N, Wei Z, Hu J (2021). Farrerol Ameliorated Cisplatin-Induced Chronic Kidney Disease Through Mitophagy Induction via Nrf2/PINK1 Pathway. Front Pharmacol.

[46] Zhou L, Zhang L, Zhang Y (2019). PINK1 Deficiency Ameliorates Cisplatin-Induced Acute Kidney Injury in Rats. Front Physiol.

[47] Huang YB, Jiang L, Liu XQ (2022). Melatonin Alleviates Acute Kidney Injury by Inhibiting NRF2/Slc7a11 Axis-Mediated Ferroptosis. Oxid Med Cell Longev.

[48] Fan X, Zhang X, Liu LC (2022). Hemopexin accumulates in kidneys and worsens acute kidney injury by causing hemoglobin deposition and exacerbation of iron toxicity in proximal tubules. Kidney Int.

[49] Lu Q, Wang M, Gui Y (2020). Rheb1 protects against cisplatin-induced tubular cell death and acute kidney injury via maintaining mitochondrial homeostasis. Cell Death Dis.

[50] Sun Y, Berleth N, Wu W (2021). Fin56-induced ferroptosis is supported by autophagy-mediated GPX4 degradation and functions synergistically with mTOR inhibition to kill bladder cancer cells. Cell Death Dis.

[51] Song X, Zhu S, Chen P (2018). AMPK-Mediated BECN1 Phosphorylation Promotes Ferroptosis by Directly Blocking System Xc(-) Activity. Curr Biol.

[52] Hou W, Xie Y, Song X (2016). Autophagy promotes ferroptosis by degradation of ferritin. Autophagy.

[53] Yang M, Chen P, Liu J (2019). Clockophagy is a novel selective autophagy process favoring ferroptosis. Sci Adv.

[54] Doherty J, Baehrecke EH (2018). Life, death and autophagy. Nat Cell Biol.

[55] Yu F, Zhang Q, Liu H (2022). Dynamic O-GlcNAcylation coordinates ferritinophagy and mitophagy to activate ferroptosis. Cell Discov.

[56] Rademaker G, Boumahd Y, Peiffer R (2022). Myoferlin targeting triggers mitophagy and primes ferroptosis in pancreatic cancer cells. Redox Biol.

[57] Basit F, van Oppen LM, Schockel L (2017). Mitochondrial complex I inhibition triggers a mitophagy-dependent ROS increase leading to necroptosis and ferroptosis in melanoma cells. Cell Death Dis.

[58] Li J, Li M, Ge Y (2022). beta-amyloid protein induces mitophagy-dependent ferroptosis through the CD36/PINK/PARKIN pathway leading to blood-brain barrier destruction in Alzheimer's disease. Cell Biosci.

[59] Li W, Xiang Z, Xing Y (2022). Mitochondria bridge HIF signaling and ferroptosis blockage in acute kidney injury. Cell Death Dis.

[60] Heyman SN, Rosen S, Silva P (1991). Protective action of glycine in cisplatin nephrotoxicity. Kidney Int.

[61] Diwan A, Krenz M, Syed FM (2007). Inhibition of ischemic cardiomyocyte apoptosis through targeted ablation of Bnip3 restrains postinfarction remodeling in mice. J Clin Invest.

[62] Kim S, Kang SW, Joo J (2021). Characterization of ferroptosis in kidney tubular cell death under diabetic conditions. Cell Death Dis.

[63] Guo C, Pei L, Xiao X (2017). DNA methylation protects against cisplatin-induced kidney injury by regulating specific genes, including interferon regulatory factor 8. Kidney Int.

[64] Zhu X, Li S, Lin Q (2021). alphaKlotho protein has therapeutic activity in contrast-induced acute kidney injury by limiting NLRP3 inflammasome-mediated pyroptosis and promoting autophagy. Pharmacol Res.

[65] Banu K, Lin Q, Basgen JM (2021). AMPK mediates regulation of glomerular volume and podocyte survival. JCI Insight.

[66] Li S, Lin Q, Shao X (2020). Drp1-regulated PARK2-dependent mitophagy protects against renal fibrosis in unilateral ureteral obstruction. Free Radic Biol Med.

