# Supplementary material for: Mitophagy alleviates cisplatin-induced renal tubular epithelial cell ferroptosis through ROS/HO-1/GPX4 axis
Source: Int J Biol Sci. 2023 Feb 13;19(4):1192–210. doi: 10.7150/ijbs.80775 (PMC10008689; doi:10.7150/ijbs.80775)
Supplement: Supplementary file 1 — Supplementary figures. [file ijbsv19p1192s1.pdf]

# FigS1 Cisplatin induced renal tubular epithelial cell ferroptosis in vivo and in vitro

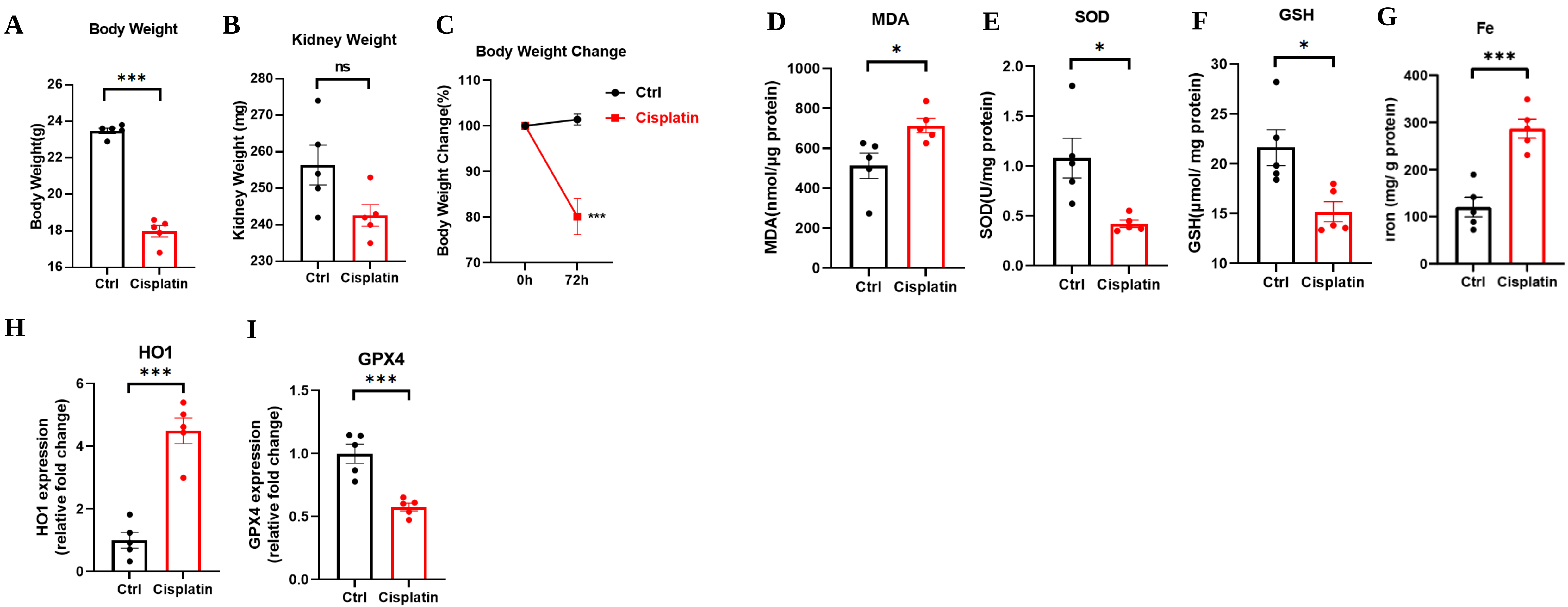


FigS1. Cisplatin induced renal tubular epithelial cell ferroptosis in vivo and in vitro.(A) The body weight and (B) kidney weight of Ctrl and Cisplatin mice. (C) The change of body weight from baseline and cisplatin treatment for 72h .(D-G) MDA, SOD,GSH and Fe level in cell homogenate were used to detect ROS, Glutathione and iron level in kidney lysates. (H,I) Immunoblotting analysis and quantification of HO1, and GPX4 in kidney lysates. Data were presented as mean ± SEM. n=3-5, ns: no significant, *p<0.05, ***p<0.001.

# FigS2 *Bnip3* knockout aggravated cisplatin-induced acute kidney injury

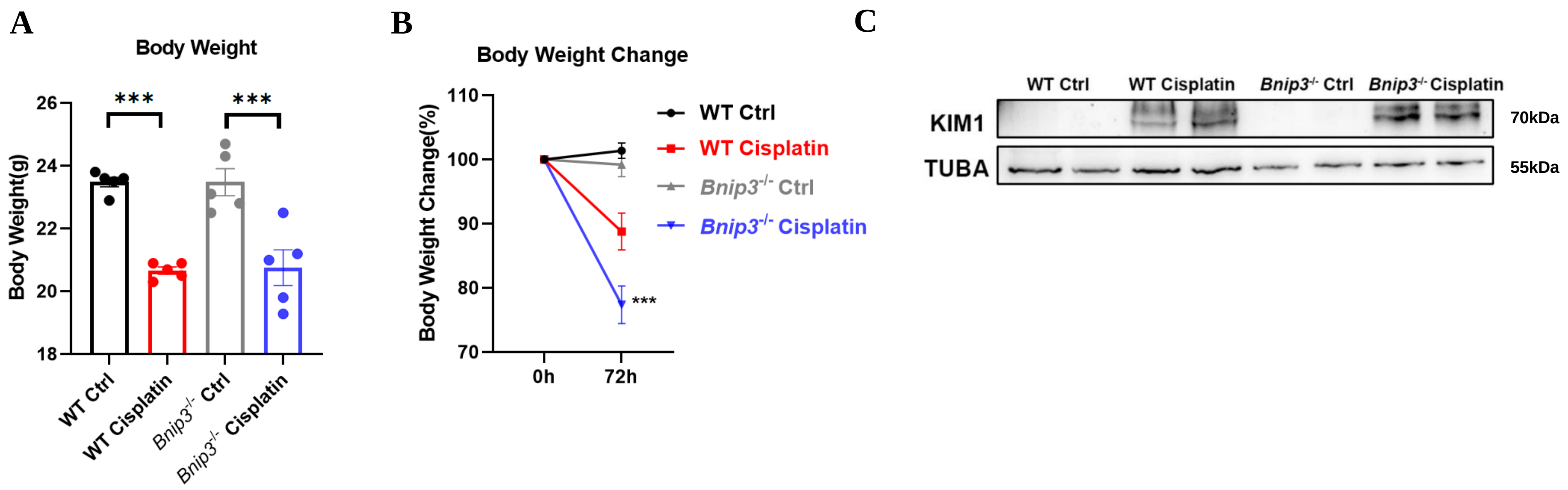


FigS2. *Bnip3* knockout aggravated cisplatin-induced acute kidney injury. (A)The body weight of mice at the prefusion time. (B) The change of body weight from baseline and cisplatin treatment for 72h. (C)The protein level of KIM1 in kidney homogenate. n=5, **p<0.01.

# FigS3 *Pink1* knockout aggravated cisplatin-induced acute kidney injury

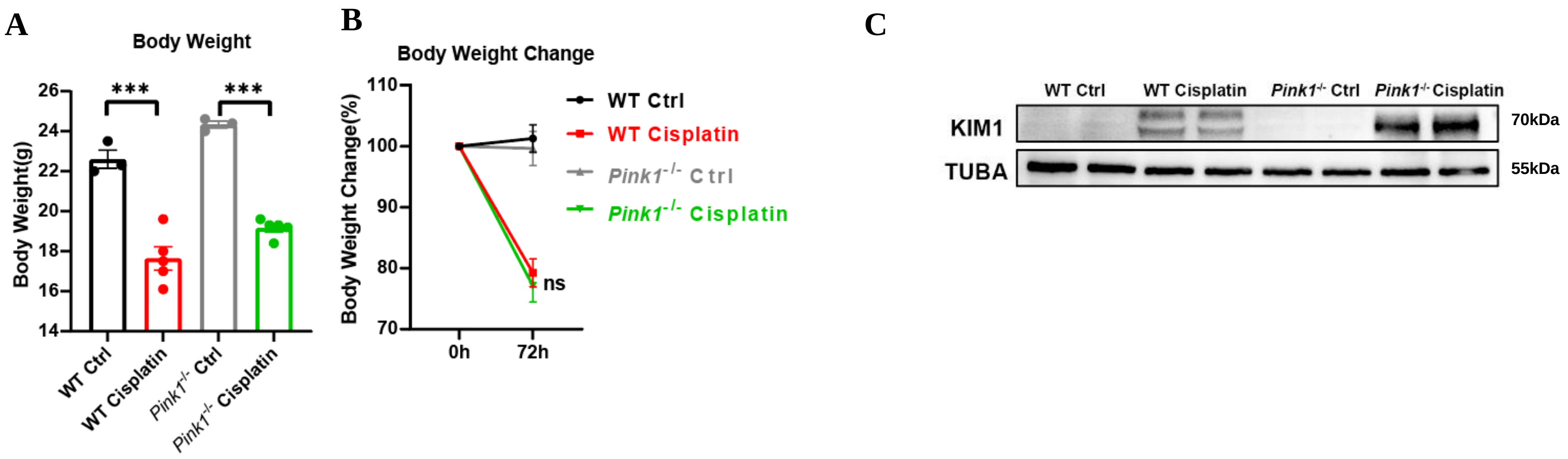


FigS3. *Pink1* knockout aggravated cisplatin-induced acute kidney injury.(A) The body weight at the prefusion time.(B) The change of body weight from baseline and cisplatin treatment for 72h.n=3-5. (C)The protein level of KIM1 in kidney homogenate. Data were presented as mean ± SEM. ns: no significant, *p<0.05, ***p<0.001.

# FigS4 *Park2* knockout aggravated cisplatin-induced acute kidney injury

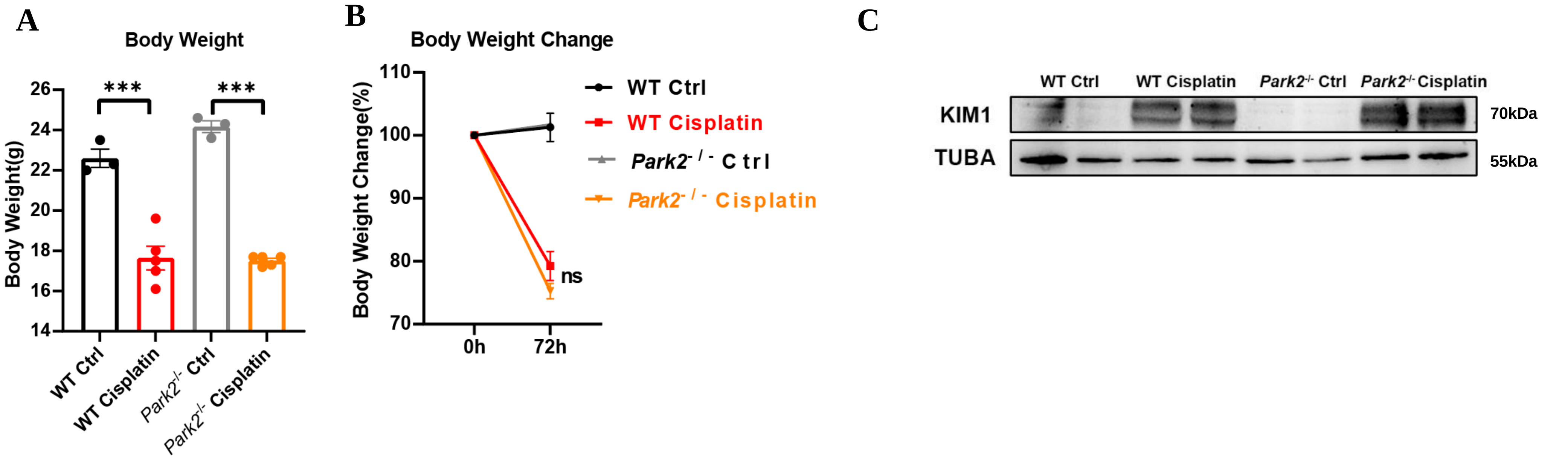


FigS4. *Park2* knockout aggravated cisplatin-induced acute kidney injury.(A) The body weight at the prefusion time.(B) The change of body weight from baseline and cisplatin treatment for 72h. (C)The protein level of KIM1 in kidney homogenate. n=3-5. Data were presented as mean ± SEM. ns: no significant, $*p<0.05$, $**p<0.01$, $***p<0.001$.
